# Comorbid epilepsy and depression—pharmacokinetic and pharmacodynamic drug interactions

**DOI:** 10.3389/fphar.2022.988716

**Published:** 2022-10-07

**Authors:** Barbara Miziak, Stanisław J. Czuczwar, Ryszard Pluta

**Affiliations:** ^1^ Department of Pathophysiology, Medical University, Lublin, Poland; ^2^ Laboratory of Ischemic and Neurodegenerative Brain Research, Mossakowski Medical Research Institute, Polish Academy of Sciences, Warsaw, Poland

**Keywords:** antiepileptic drugs (AEDs), antidepressant drugs, pharmacodynamic interactions, phrmacokinetic interactions, seizures, epilepsy, depression

## Abstract

**Background:** Major depressive disorder may be encountered in 17% of patients with epilepsy and in patients with drug-resistant epilepsy its prevalence may reach 30%. This indicates that patients with epilepsy may require antidepressant treatment.

**Purpose:** Both pharmacodynamic and pharmacokinetic interactions between antiepileptic (antiseizure) and antidepressant drugs have been reviewed. Also, data on the adverse effects of co-administration of antiepileptic with antidepressant drugs have been added. This article was submitted to Neuropharmacology, a section of the journal Frontiers in Pharmacology.

**Methods:** The review of relevant literature was confined to English-language publications in PUBMED databases. Table data show effects of antidepressants on the seizure susceptibility in experimental animals, results of pharmacodynamic interactions between antiepileptic and antidepressant drugs mainly derived from electroconvulsions in mice, as well as results concerning pharmacokinetic interactions between these drugs in clinical conditions.

**Conclusion:** Antidepressant drugs may exert differentiated effects upon the convulsive threshold which may differ in their acute and chronic administration. Animal data indicate that chronic administration of antidepressants could reduce (mianserin, trazodone) or potentiate the anticonvulsant activity of some antiepileptics (fluoxetine, reboxetine, venlafaxine). There are also examples of neutral interactions (milnacipran).

## 1 Introduction

The prevalence for major depressive disorder (MDD) is in the range of 10% in the general adult population but as a comorbid condition in epilepsy, MDD is observed more frequently. For instance, it is observed in around 17% of patients with epilepsy and in patients with drug-resistant epilepsy, its prevalence may reach 30% or even more. Interestingly, in patients with remissions, MDD is noted in 6–9% of cases (Wiegartz et al., 1999; Jackson and Turkington, 2005; Tellez-Zenteno et al., 2007; Piedad et al., 2012). Depression is evidently responsible for the worsening of life quality in patients with epilepsy and may additionally increase seizure frequency and reduce the anticonvulsant efficacy of antiepileptic drugs (Hitiris et al., 2007).

A question arises whether depression may be a significant risk factor for the development of epilepsy. The existing evidence seems to confirm this possibility and there are even opinions available, pointing to common pathophysiological mechanisms in depression and epilepsy (Kanner, 2008). Both, in patients with temporal lobe epilepsy or depression, an enhanced hippocampal interleukin-1β signaling was evident (Kondziella et al., 2007). The involvement of reduced GABA-mediated inhibition and excessive glutamatergic neurotransmission are, no doubt, involved in generation of seizure activity (Łukawski et al., 2016). Disturbed GABA-ergic and glutamatergic neurotransmissions have been also observed in depression, for instance proton magnetic resonance spectroscopy revealed abnormal GABA and glutamate concentrations in the cortex and glutamate receptor antagonists were shown to exert antidepressant effects in animal models (Brambilla et al., 2003; Kugaya and Sanacora, 2005). After all, both epilepsy and depression are accompanied by atrophy of the temporal lobe (amygdalar nuclei, hippocampus, entorhinal cortex), frontal lobe, and temporal lateral neocortex. Also, reduced binding of serotonin in the frontal and temporal lobes, clear cut dysfunction of the hypothalamic-pituitary-adrenal axis, altered tryptophan metabolism, dysregulated neurogenesis, and neuroinflammation were found (Kanner, 2008; Singh and Goel, 2021).

As already mentioned, the prevalence of MDD in patients with epilepsy is high and surprisingly, in many cases depression is not diagnosed and remains untreated, possibly because of fear of adverse effects that might be induced by antidepressant drugs and their probable negative influence upon the convulsive threshold (Kanner et al., 2012; Bosak et al., 2015; Gangar and Bhatt, 2020; Singh and Goel, 2021). There are factors, evidently increasing the risk of depression in patients with epilepsy–older age, female gender, low level of education, unemployment, poor pharmacological control of seizures, polytherapy, stigma, and anxiety (Yang et al., 2020). The female gender faces a higher risk of depression in patients with epilepsy, preferably during the reduction of estrogenic activity encountered in the post-partum and late luteal phase. The estrogenic reduction may be associated with up-regulation of single nucleotide polymorphisms, and down-regulation of serotonin- and GABA-mediated events (Zarcone and Corbetta, 2017).

Below, the influence of a number of antidepressant drugs upon the seizure susceptibility of experimental animals in a wide range of seizure models as well as pharmacodynamic interactions between antiepileptic and antidepressant drugs are reviewed. Review of clinical data will be focused on pharmacokinetic interactions between these drugs. Literature search was performed basing mainly on PUBMED databases and English-language publications with no time frame. Relevant references were also considered from extracted publications.

## 2 Antidepressant drugs and seizure susceptibility

In many cases, the influence antidepressant drugs on the seizure threshold refers to either acute or chronic dosing. The results are presented in Table 1 on the basis of data reviewed by Banach et al. (2016).

**TABLE 1:** Influence of various antidepressant drugs on the seizure threshold and epileptiform activity.

Class of antidepressants	Antidepressant	Dose of antidepressant (mg/kg)	Treatment		Method of administration	Animal model	Effect on seizures	
			Acute	Chronic			Epileptiform activity	Seizure threshold
Tricyclic antidepressants	Amitriptyline	20–50	+		i.p. or i.v	Rabbits	↑	
						Cats		
						Rats (implanted with intracerebral electrodes/kindling)		
						Pilocarpine-induced seizures in rats	↑	
		20–30	+		i.p	Mice (electrically induced convulsions)		↑
	Imipramine	1–25	+		i.v	Rabbits	↑	
						Cats implanted with intracerebral electrodes freely moving rats		
		10–50	+		i.p	Mice		
						Photosensitive baboons (papio papio)		
						Sham and mygdale-kindled rats		
		≥20–25	+		i.p	Cats	↑	
						Mice		
		30–40	+		i.p	Mice (electrically induced convulsions))		↑
	Desipramine	NNo data	No data	No data	No data	Freely moving rats implanted with intracerebral electrodes	↑	
						Mice (flurothyl-induced seizures)		↓
						Rats (kindling)	↓	
						Rats (audiogenic seizures in genetically epilepsy-prone	↓	
		up to 40				Mice		0
Selective serotonin reuptake inhibitors	Fluoxetine	5–20	+		No data	Mice	protective effect	
		10	+			Rats	↑	
		2,5-20	+			Rats	0	
		10	+			Rats (PTZ-induced seizures)		0
		10–20	+		i.p	Rats (seizures induced by pilocarpine)	↑ or ↓	
		ED_50_ = 15.9 or 8.2	+		No data	Wistar rats with high predisposition to audiogenic seizures (sound-induced seizures in (geprs))	↓	
		2.5–10	+		i.p	Rat model of ethanol withdrawal syndrome	↓	
		5–20	+		applied into substantia nigra or i.p	Rats (seizures evoked by injection of bicuculline into the area tempestas)	↓	
		15–25	+		No data	Mice (MES induced seizures)		↑
		10 (chronic treatment, 7 days)		+	No data	Rats (PTZ-induced seizures)		↓
				+	s.c	Amygdala-kindled rats	↑	
		10		+	Dietary	Mouse model of genetically predisposed/handling-triggered epilepsy	↓	
		10		+	No data	Rats (electrically induced hippocampal seizures)		↑
		10	+			Mice (MES induced seizures)		0
				+				
	Fluoxetine + 5-Hydroxytrypto-phan	15	+		i.p	Rats (sound-induced seizures in (geprs))	↓	
	Paroxetine	1	+		No data	Mice (picrotoxin induced Seizures)	↓	
		50	+		No data	Rats (sham and amygdala-kindled)	No effect in behavior or EEG	
	Sertraline	40/24 h		+	via osmotic minipump	Mice (flurothyl-induced seizures)	0	
		40	+					↓
		2.5	+		No data	Rats (4-aminopyridine seizures)	↓	
		0.75/day		+				
		>5	+		No data	Rats (PTZ-induced seizures)	↓	
Serotonin and norepinephrine reuptake inhibitors	Venlafaxine	20–40	+		i.p	Audiogenic seizures in the rat model of ethanol withdrawal syndrome	↓	
			+			Mice (flurothylinduced seizures)		
		75–100	+			Rats (PTZ-induced seizures)	↑	
		150	+			Rats (spontaneous convulsions)	↑	
		12.5–25	+	+		Mice (electrically induced convulsions)		↑
	Milnacipran	10–40	+		i.p	Mice (electrically induced convulsions)		↑
Noradrenaline reuptake inhibitor	Reboxetine	20		+	via osmotic minipumps	Mice (flurothyl-induced seizures)	↑ ↓	↓
		20–30	+		No data	Kainic acid-induced post-status epilepticus rat model for temporal lobe epilepsy	↓	
			+		No data	Zebrafish larva (PTZ-induced seizures)	↓	
		10–20	+		No data	Mice (PTZ-induced seizures)		↑
		30	+		No data	Mice (pilocarpine-induced limbic seizures)	↑	
		8–16	+		No data	Mice (electrically induced convulsions)		↑
				+				0
Norepinephrine and dopamine reuptake inhibitor	Bupropion	ED_50_–19.4	+		No data	Mice (MES-induced seizures)	↓	
		CD97–139.5	+		No data	Seizure activity per se	↑	
		7.5–10 twice daily		+	No data	Mice (electrically induced convulsions)		↑
		10 and 50	+		No data	Rats (kainic acid-induced seizures)	↓	
Reversible inhibitor of monoamine oxidase A	Moclobemide	62.5–75	+		No data	Mice (electrically induced convulsions)		↑
Other antidepressants	Mianserin	81.2	+		i.v	Rabbits	↑	
		30	+		i.v	Rats (implanted with intracerebral electrodes	↑	
		30–40	+		No data	Mice (electrically induced convulsions)		↑
	Trazodone	10–40	+		No data	Mice (electrically induced convulsions)		0
		40		+	No data			↑
		up to 80	+		No data	PTZ-, strychnine-, imipramine- and MES-induced seizures	0	
		100					↑	
	Tianeptine	1.25–10	+		No data	Rats (PTZ-induced seizures)	↓	
		40–80	+		No data	Mice (PTZ-induced seizures)	↓	
		5–20	+		i.p	Audiogenic seizures in the rat model of ethanol withdrawal syndrome	↓	
				+				
		25–50	+		No data	Mice (electrically induced convulsions)		0
				+				

↑—Increase in convulsive activity/increase of the convulsion threshold; ↓—decrease in convulsive activity/decrease of the convulsion threshold; 0–no impact; MES, maximal electroshock test; PTZ, pentylenetetrazol; i.p, intraperitoneal injection; i.v.–intravenous injection; s.c.–subcutaneous administration. Data taken from Banach et al. (2016).

## 3 Interactions between antiepileptic and antidepressant drugs in animal seizure models

### 3.1 Selective serotonin reuptake inhibitors

Fluoxetine (given acutely at subprotective doses against electroconvulsions in mice) potentiated the anticonvulsant activity of a number of conventional AEDs (carbamazepine, phenobarbital or phenytoin) against maximal electroshock-induced seizures (MES) in mice (Leander, 1992; Borowicz et al., 2006). In the same seizure test, fluoxetine (but at higher dose of 15–25 mg/kg) was also able to increase the protective action of valproate (Borowicz et al., 2006). Pharmacokinetic verification revealed that the brain concentrations of carbamazepine and phenobarbital were elevated (Borowicz et al., 2006). When administered chronically, this SSRI (at 15–20 mg/kg) was effective in enhancing the antiseizure efficacy of carbamazepine, phenytoin, and valproate whilst the protective activity of phenobarbital was increased only at 20 mg/kg of fluoxetine (Borowicz et al., 2007b). The brain concentrations of all conventional antiepileptic drugs studied were significantly increased by fluoxetine (Borowicz et al., 2007b).

Considering pentylenetetrazol (PTZ)-induced seizures in mice, acute fluoxetine (10 mg/kg) potentiated the anticonvulsant action of valproate whilst it was ineffective when combined with ethosuximide. Brain concentration of valproate was not affected and co-administered fluoxetine did not augment the neurotoxic potential of either valproate or ethosuximide (Borowicz et al., 2012b).

Sertraline (given orally at 10 mg/kg to mice) significantly reduced the protective efficacy of gabapentin against PTZ which manifested in a considerable increase in seizure severity (Rizwan et al., 2003). Interestingly, sertraline at this dose also exacerbated PTZ-induced convulsions (Rizwan et al., 2003).

As regards acute paroxetine, this SSRI (1 mg/kg) enhanced the anticonvulsant activity of valproate (50 mg/kg) reflected by the prolonged latency to onset of seizures induced by picrotoxin (3.5 mg/kg) in mice. The evident reduction of the seizure score by valproate was not further potentiated by paroxetine (Kamal, 2012). The author of this study evaluated some more parameters. The results indicate that valproate-produced elevation of GABA within nucleus accumbens was further significantly increased by paroxetine (Kamal, 2012).

### 3.2 Serotonin and noradrenaline reuptake inhibitors

In the MES test, acute milnacipran (at 10 mg/kg, which elevated the convulsive threshold) reduced the ED_50_ values of conventional antiepileptic drugs (carbamazepine, phenobarbital, phenytoin, valproate) whilst at a subthreshold dose of 5 mg/kg, only the antiseizure activity of phenobarbital and carbamazepine was enhanced (Borowicz et al., 2010). Interestingly, chronic milnacipran (5–40 mg/kg) remained ineffective upon the protection offered by these antiepileptic drugs. No pharmacokinetic interactions with antiepileptic drugs, regarding acute or chronic milnacipran, were noted. Milnacipran did not also potentiate the neurotoxic potential of the conventional antiepileptics (Borowicz et al., 2010).

Venlafaxine was evaluated in the same convulsive test. At the subthreshold single or chronic dose of 6.25 mg/kg, this SNRI elevated the protective action of valproate. At 12.5 mg/kg (the dose increasing the convulsive threshold), venlafaxine was also able to potentiate the antiseizure activity of carbamazepine and phenobarbital but not that of phenytoin (Borowicz et al., 2011). The brain concentration of phenytoin was lowered by venlafaxine so the enhancing effect of venlafaxine could be masked by a pharmacokinetic interaction (Borowicz et al., 2011).

### 3.3 Noradrenaline reuptake inhibitor

Reboxetine (in acute doses of 2–8 mg/kg) combined with carbamazepine, phenobarbital and valproate led to reductions in their ED_50_ against MES in mice. Potentiation of the anticonvulsant action of phenytoin was observed for reboxetine at the dose range of 8–12 mg/kg. Importantly, this antidepressant drug elevated the electroconvulsive threshold starting from 8 mg/kg (Borowicz et al., 2014). When given chronically, this NRI (8–12 mg/kg) enhanced the protection by CBZ against MES, being, however, ineffective when combined with phenobarbital, phenytoin or valproate. The antidepressant drug, following chronic administration, did not affect the electroconvulsive threshold. In neither effective combination of reboxetine with antiepileptic drugs pharmacokinetic interactions were observed in terms of the brain concentration of these drugs (Borowicz et al., 2014).

Regarding PTZ-induced convulsions in mice, acute reboxetine, in the subthreshold doses of 6–8 mg/kg, did not affect the protective efficacy of clonazepam, ethosuximide, phenobarbital and valproate (Popławska et al., 2015).

### 3.4 Noradrenaline and dopamine reuptake inhibitor

Bupropion, similarly to some antiepileptic drugs, was acutely effective against MES in mice with an ED_50_ value of 19.4 mg/kg. In doses exceeding 100 mg/kg, this antidepressant proved as a convulsive agent (Tutka et al., 2004). When given chronically to mice at 5 mg/kg, the NDRI significantly enhanced the anticonvulsant effect of felbamate, lamotrigine and topiramate against MES. However, the combined treatment with lamotrigine resulted in a pharmacokinetic interaction as both, the plasma and brain level of this antiepileptic drug were significantly elevated. Although the plasma concentration of topiramate was reduced, its brain concentration was unaffected by bupropion. Neurotoxicity of antiepileptic drugs was not influenced by the antidepressant drug (Barczyński et al., 2011).

### 3.5 Reversible inhibitor of monoamine oxidase A

Acute but not chronic moclobemide (62.5 and 75 mg/kg) raised the threshold for electroconvulsions in mice. When given acutely, moclobemide (up to the subthreshold dose of 50 mg/kg) potentiated the anti-MES efficacy of carbamazepine, phenobarbital and valproate. Chronic RIMA (37.5–75 mg/kg) enhanced the protection offered by carbamazepine, phenobarbital, phenytoin and valproate against MES in mice and only its combination with phenobarbital was of pharmacodynamic nature. Its other combinations with antiepileptic drugs resulted in significant elevations of their brain concentrations. However, neither acute nor chronic moclobemide co-administered with antiepileptic drugs produced any impairment of motor performance or deficit of long-term memory (Borowicz-Reutt and Banach, 2021).

### 3.6 Tricyclic antidepressant drugs

Amitriptyline (at a single dose of 20 or 30 mg/kg) elevated the electroconvulsive threshold in mice and at the subthreshold dose of 10 mg/kg, it potentiated the anticonvulsant action of valproate against MES in mice. The ED_50_ value of valproate was diminished from 255 to 150 mg/kg (Kleinrok et al., 1991), Desipramine (acutely up to 40 mg/kg) was ineffective upon electroconvulsions in mice but at 20 mg/kg, the antidepressant significantly reduced the ED_50_ of valproate against MES from 255 to 135 mg/kg (Kleinrok et al., 1991).

As regards imipramine, the antidepressant given acutely at 30 or 40 mg/kg proved effective against electroconvulsions and at 20 mg/kg potentiated the protective action of valproate against MES in mice reflected by the reduction of its ED_50_ from 255 to 128 mg/kg (Kleinrok et al., 1991). No pharmacokinetic interactions were evident in combinations of valproate with desipramine. Other combinations of valproate with TCAs were not verified in terms of pharmacokinetics. Also, data on the interactions of chronic TCAs and AEDs are not available.

### 3.7 Other antidepressant drugs

Mianserin administered acutely (30 and 40 mg/kg) significantly raised the electronvulsive threshold in mice but when given chronically at 30 mg/kg, it was proconvulsant in the threshold electoconvulsive test (Borowicz et al., 2007a). Combinations of acute mianserin (up to 20 mg/kg) with antiepileptic drugs resulted in the reductions of ED_50_ values of carbamazepine, phenytoin and valproate against MES in mice. In contrast, mianserin given chronically (up to 20 mg/kg) decreased the anticonvulsant activity of phenytoin and valproate against MES whilst the protective action of carbamazepine against MES was unaffected. Modifications of the anticonvulsant efficacy of antiepileptic drugs by mianserin was of pharmacodynamic nature. Neither combination was associated with neurotoxicity evaluated in the chimney test (motor coordination) and passive avoidance task (long-term memory) (Borowicz et al., 2007a).

Tianeptine (25–50 mg/kg) neither acutely nor chronically affected the threshold for electroconvulsions in mice. In combinations with antiepileptic drugs, acute and chronic tianeptine (up to 50 mg/kg) potentiated the protective action of carbamazepine, phenobarbital and valproate, but not that of phenytoin, in the mouse MES test (Borowicz et al., 2013). Only the brain concentration of phenobarbital was reduced by tianeptine, other combinations showing pharmacodynamic profile. Motor coordination and long-term memory were not impaired in mice by combinations of tianeptine with antiepileptic drugs (Borowicz et al., 2013).

Trazodone, given singly at 10–40 mg/kg did not modify the electroconvulsive threshold in mice whilst on a chronic basis at 40 mg/kg it raised the threshold (Borowicz et al., 2012a). The acute antidepressant (up to 40 mg/kg), significantly reduced the anticonvulsant efficacy of carbamazepine and phenytoin against mouse MES. The protective action of phenobarbital and valproate in this test was not changed when combined with trazodone (up to 40 mg/kg). Combinations of chronic trazodone (up to 40 mg/kg) with antiepileptic drugs in the above test, yielded comparable results to its acute administration. Combined treatments of trazodone with antiepileptic drugs resulted in numerous pharmacokinetic interactions. The brain level of phenytoin was decreased and that of valproate elevated when co-administered with acute or chronic trazodone. Only chronic trazodone reduced the brain concentration of carbamazepine and phenobarbital. Finally, acute and chronic trazodone significantly augmented the neurotoxicity of phenytoin but not that of other antiepileptic drugs (Borowicz et al., 2012a). Detailed results of interactions between AEDs and antidepressants in seizure models have been presented in Table 2.

**TABLE 2:** Interactions between AEDs and antidepressant drugs in experimental seizure models.

Class of antidepressants	Antidepressant	Dose of antidepressant [mg/kg]	Treatment		Method of administration	Antiepileptic drug	Dose of AEDs/ED_50_^c^ ED_50_^e^ (mg/kg)	Method of administration	Seizure model	Anticonvulsant activity of AEDs	Neurotoxic effects of AEDs				References
			Acute	Chronic							Chimney test	Passive-avoidance task	Rotarod test	Spontaneous alternation behavior (SAB) models	
Tricyclic antidepressants	Amitriptyline	10	+		i. p	VPA	ED_50_^c^–255/ED_50_^e^–150	i. p	MES	↑	-	-	-	-	Kleinrok et al. (1991)
	Imipramine	20	+		i. p	VPA	ED_50_^c^–255/ED_50_^e^–128	i. p	MES	↑	-	-	-	-	
	Desipramine	20	+		i. p	VPA	ED_50_^c^–255/ED_50_^e^–135	i. p	MES	↑	-	-	-	-	Kleinrok et al. (1991)
Selective serotonin reuptake inhibitors	Fluoxetine	15–20		+	i. p	CBZ	ED_50_^c^–9.8/ED_50_^e^–4.1	i.p	MES	↑	0	0	-	-	Borowicz et al. (2007a)
	Fluoxetine	15–20		+	i.p	PHT	ED_50_^c^–12.2/ED_50_^e^–7.6	i.p	MES	↑	0	0	-	-	Borowicz et al. (2007b)
	Fluoxetine	15–20		+	i.p	VPA	ED_50_^c^–211/ED_50_^e^–130.6	i.p	MES	↑	0	0	-	-	Borowicz et al. (2007a)
	Fluoxetine	20		+	i.p	PB	ED_50_^c^–25.5/ED_50_^e^–18.5	i.p	MES	↑	0	0	-	-	Borowicz et al. (2007b)
	Fluoxetine	10	+		i.p	VPA	ED_50_^c^ –117.2/ED_50_^e^–78.2	i.p	PTZ	↑	0	0	-	-	Borowicz et al. (2012a)
	Fluoxetine	10	+		i.p	ETX	ED_50_^c^–137.7/ED_50_^e^–122.1	i.p	PTZ	0	0	0	-	-	Borowicz et al. (2012b)
	Fluoxetine	15–25	+		i.p	VPA	ED_50_^c^–212.5/ED_50_^e^–151.1–110.3	i.p	MES	↑	0	0	-	-	Borowicz et al. (2006)
	Paroxetine	1	+		i.p	VPA	50	i.p	seizures induced by picrotoxin	↑	-	-	-	-	Kamal (2012)
	Sertraline	10	+		p.o	GBP	312	p.o	PTZ	↓	-	-	-	↓	Rizwan et al. (2003)
Serotonin and norepinephrine reuptake inhibitors	Venlafaxine	6.25	+		i.p	PHT	ED_50_–10	i.p	MES	0	0	0	-	-	Borowicz et al. (2011)
	Venlafaxine	6.25	+		i.p	VPA	ED50c –253.5/ED50e–199.6	i.p	MES	↑	0	0	-	-	Borowicz et al. (2011)
	Venlafaxine	6.25		+	i.p	VPA	ED_50_^c^–210.7/ED_50_^e^–142.6	i.p	MES	↑	0	0	-	-	Borowicz et al. (2011)
	Venlafaxine	12.5	+		i.p	CBZ	ED_50_^c^–10.3/ED_50_^e^–6.7	i.p	MES	↑	0	0	-	-	Borowicz et al. (2011)
	Venlafaxine	12.5	+		i.p	PB	ED_50_^c^–15.4/ED_50_^e^–10.7	i.p	MES	↑	0	0	-	-	Borowicz et al. (2011)
	Venlafaxine	12.5	+		i.p	PHT	ED_50_^c^–12.4/ED_50_^e^–6.4	i.p	MES	0	0	0	-	-	Borowicz et al. (2011)
	Milnacipran	10	+		i.p	CBZ	ED_50_^c^–11.2/ED_50_^e^–5.2	i.p	MES	↑	0	0	-	-	Borowicz et al. (2010)
	Milnacipran	10	+		i.p	PB	ED_50_^c^–18.4/ED_50_^e^–14.4	i.p	MES	↑	0	0	-	-	Borowicz et al. (2010)
	Milnacipran	10	+		i.p	PHT	ED_50_^c^–8.5/ED_50_^e^–5.1	i.p	MES	↑	0	0	-	-	Borowicz et al. (2010)
	Milnacipran	10	+		i.p	VPA	ED_50_^c^–213/ED_50_^e^–157.1	i.p	MES	↑	0	0	-	-	Borowicz et al. (2010)
	Milnacipran	5	+		i.p	CBZ	ED_50_^c^–11.2/ED_50_^e^–6.2	i.p	MES	↑	0	0	-	-	Borowicz et al. (2010)
	Milnacipran	5	+		i.p	PB	ED_50_^c^–18.4/ED_50_^e^–11.9	i.p	MES	↑	0	0	-	-	Borowicz et al. (2010)
	Milnacipran	5–40		+	i.p	CBZ	ED_50_^c^–11.2/ED_50_^e^–10.1	i.p	MES	0	0	0	-	-	Borowicz et al. (2010)
	Milnacipran	5–40		+	i.p	PB	ED_50_^c^–18.4/ED_50_^e^–15.1	i.p	MES	0	0	0	-	-	Borowicz et al. (2010)
	Milnacipran	5–40		+	i.p	PHT	ED_50_^c^–8.5/ED_50_^e^–10.6	i.p	MES	0	0	0	-	-	Borowicz et al. (2010)
	Milnacipran	5–40		+	i.p	VPA	ED_50_^c^–213/ED_50_^e^–210.7	i.p	MES	0	0	0	-	-	Borowicz et al. (2010)
Noradrenaline reuptake inhibitor	Reboxetine	6–8	+		i.p	CLZ	ED_50_^c^–0.0019/ED_50_^e^–0.014	i.p	PTZ	0	0	0	-	-	Popławska et al. (2015)
		6–8	+		i.p	ETX	ED_50_^c^–140.9/ED_50_^e^–139.6	i.p	PTZ	0	0	0	-	-	Popławska et al. (2015)
		6–8	+		i.p	PB	ED_50_^c^–18/ED_50_^e^–15.3	i.p	PTZ	0	0	0	-	-	Popławska et al. (2015)
		6–8	+		i.p	VPA	ED_50_^c^–115.3/ED_50_^e^–101.6	i.p	PTZ	0	0	0	-	-	Popławska et al. (2015)
		2–8	+		i.p	CBZ	ED_50_^c^–11.3/ED_50_^e^–7.2	i.p	MES	↑	0	0	-	-	Borowicz et al. (2014)
		2–8	+		i.p	PB	ED_50_^c^–18.7/ED_50_^e^–11.9	i.p	MES	↑	0	0	-	-	Borowicz et al. (2014)
		2–8	+		i.p	VPA	ED_50_^c^–244.3/ED_50_^e^–181	i.p	MES	↑	0	0	-	-	Borowicz et al. (2014)
		8–12	+		i.p	PHT	No data	i.p	MES	↑	0	0	-	-	Borowicz et al. (2014)
		8–12		+	i.p	CBZ	ED_50_^c^ –11.9/ED_50_^e^–8.7	i.p	MES	↑	0	0	-	-	Borowicz et al. (2014)
		8–12		+	i.p	PB	ED_50_^c^–24.1/ED_50_^e^–22.7	i.p	MES	0	0	0	-	-	Borowicz et al. (2014)
		8–12		+	i.p	PHT	ED_50_^c^–9.9/ED_50_^e^–9.4	i.p	MES	0	0	0	-	-	Borowicz et al. (2014)
		8–12		+	i.p	VPA	ED_50_^c^–240.2/ED_50_^e^–203.1	i.p	MES	-	0	0	-	-	Borowicz et al. (2014)
Norepinephrine and dopamine reuptake inhibitor	Bupropion	5		+	i.p	Felbamate	ED_50_^c^–48.79/ED_50_^e^–37.28	i.p	MES	↑	-	-	0	-	Barczyński et al. (2011)
				+	i.p	LTG	ED_50_^c^–4.58/ED_50_^e^–3.01	i.p	MES	↑	-	-	0	-	
				+	i.p	TPM	ED_50_^c^–60.95/ED_50_^e^–41.58	i.p	MES	↑	-	-	0	-	
Reversible inhibitor of monoamine oxidase A	Moclobemide	50	+		i.p	CBZ	ED_50_^c^–9.9/ED_50_^e^–2.9	i.p	MES	↑	0	0	-	-	Borowicz-Reutt and Banach (2021)
		50	+		i.p	PB	ED_50_^c^–16.4/ED_50_^e^–7.2	i.p	MES	↑	0	0	-	-	Borowicz-Reutt and Banach (2021)
		50	+		i.p	VPA	ED_50_^c^–211.9/ED_50_^e^–148.5	i.p	MES	↑	0	0	-	-	Borowicz-Reutt and Banach (2021)
		37.5–75		+	i.p	PHT	ED_50_^c^–9.9/ED_50_^e^–5.8	i.p	MES	↑	0	0	-	-	Borowicz-Reutt and Banach (2021)
Other antidepressants	Mianserin	up to 20	+		i.p	CBZ	ED_50_^c^ –13.2/ED_50_^e^–6.2	i.p	MES	↑	0	0	-	-	Borowicz et al. (2007a)
	Mianserin	up to 20	+		i.p	PHT	ED_50_^c^–8.3/ED_50_^e^–5.2	i.p	MES	↑	0	0	-	-	Borowicz et al. (2007b)
	Mianserin	up to 20	+		i.p	VPA	ED_50_^c^–221/ED_50_^e^–168.6	i.p	MES	↑	0	0	-	-	Borowicz et al. (2007a)
	Mianserin	up to 20	+		i.p	PHT	ED_50_^c^–7.8/ED_50_^e^–11.5	i.p	MES	↓	0	0	-	-	Borowicz et al. (2007a)
	Mianserin	up to 20	+		i.p	VPA	ED_50_^c^–244.3/ED_50_^e^–324.2	i.p	MES	↓	0	0	-	-	Borowicz et al. (2007b)
	Trazodone	20		+	i.p	CBZ	ED50c–12.5/ED50e–17.2	i.p	MES	↓	0	0	-	-	Borowicz et al. (2012a)
	Trazodone	up to 40	+		i.p	CBZ	ED50c–11.4/ED50e–15.4	i.p	MES	↓	0	0	-	-	Borowicz et al. (2012b)
	Trazodone	20		+	i.p	PHT	ED50c–9.9/ED50e–16.2	i.p	MES	↓	↑	↑	-	-	Borowicz et al. (2012a)
	Trazodone	up to 40	+		i.p	PHT	ED50c–8.2/ED50e–12.5	i.p	MES	↓	↑	↑	-	-	Borowicz et al. (2012b)
	Trazodone	20		+	i.p	PB	ED50c–25.0	i.p	MES	0	0	0	-	-	Borowicz et al. (2012a)
	Trazodone	up to 40	+		i.p	PB	ED50c–17.9	i.p	MES	0	0	0	-	-	Borowicz et al. (2012b)
	Trazodone	20		+	i.p	VPA	ED50c–245.0	i.p	MES	0	0	0	-	-	Borowicz et al. (2012a)
	Trazodone	up to 40	+		i.p	VPA	ED50c–234.0	i.p	MES	0	0	0	-	-	Borowicz et al. (2012b)
	Tianeptine	up to 50	+		i.p	CBZ	ED_50_^c^–16.6/ED_50_^e^–10.2	i.p	MES	↑	0	0	-	-	Borowicz et al. (2013)
	Tianeptine	up to 50	+		i.p	PB	ED_50_^c^–24.7/ED_50_^e^–14.2	i.p	MES	↑	0	0	-	-	Borowicz et al. (2013)
	Tianeptine	up to 50	+		i.p	VPA	ED_50_^c^–265.4/ED_50_^e^–192	i.p	MES	↑	0	0	-	-	Borowicz et al. (2013)
	Tianeptine	up to 50	+		i.p	PHT	no data	i.p	MES	0	0	0	-	-	Borowicz et al. (2013)
	Tianeptine	up to 50		+	i.p	CBZ	ED_50_^c^–19.9/ED_50_^e^–9.2	i.p	MES	↑	0	0	-	-	Borowicz et al. (2013)
	Tianeptine	up to 50		+	i.p	PB	ED_50_^c^–21.4/ED_50_^e^–16.7	i.p	MES	↑	0	0	-	-	Borowicz et al. (2013)
	Tianeptine	up to 50		+	i.p	VPA	ED_50_^c^–274.1/ED_50_^e^–194.4	i.p	MES	↑	0	0	-	-	Borowicz et al. (2013)
	Tianeptine	up to 50		+	i.p	PHT	no data	i.p	MES	0	0	0	-	-	Borowicz et al. (2013)

↑- increased; ↓—decreased;—no test was carried out (in neurotoxic effects of AEDs); 0–no effect; AEDs, antiepileptic drugs; CLZ, clonazepam; CBZ, carbamazepine; ED_50_^c^–ED_50_ in control group; ED_50_^e^–ED_50_ in experimental group; ETX, ethosuximide; GBP, gabapentin; i.p.–intraperitoneal injection; p.o.–oral administration; LTG, lamotrigine; MES, maximal electroshock-induced convulsions; PB, phenobarbital; PHT, phenytoin; PTZ, pentylenetetrazol-induced seizures; TPM, topiramate VPA, valproate.

## 4 Pharmacokinetic interactions between antiepielptic and antidepressant drugs in clinical conditions

There are many inducers of hepatic microsomal enzymes or uridine diphosphate glucuronosyltransferase (UGT) among conventional antiepileptic drugs. The hepatic cytochrome P450 system (CYP) plays a more important role in these interactions (for review, Spina et al., 2016). Apart from the liver cytochrome P450 (CYP) enzymes, also brain CYP enzymes may participate in the local metabolism of a variety of drugs and neuroactive endogenous substances (for instance, neurosteroids), being engaged in biosynthesis of neurotranmitters as well (Daniel et al., 2022). Further, the central nervous system is apparently involved, via neuroendocrine and neuroimmune mechanisms, in the adjustment of liver CYP activity. Some antidepressant drugs have been documented to affect liver and brain CYP enzymes. A good example is fluoxetine which inhibits CYP2D in the liver, striatum and nucleus accumbens whilst stimulating its activity in the cerebellum. These complex interrelations clearly indicate that *in vitro* data on CYP enzyme inhibition (Danek et al., 2020) or stimulation by a psychotropic drug (Danek et al., 2021) may be not sufficient to predict pharmacokinetic interactions with concomitant drugs. *In vivo* data may comprise all mechanisms involved in the final effect of a drug upon the activity of CYP enzymes (Daniel et al., 2022).

Potent inducers of liver CYP encompass for example, carbamazepine, phenobarbital or phenytoin which activate a number of CYP isoforms—CYP1A2, CYP2C9, CYP2C19, and CYP3A4 (Spina et al., 2016). Consequently, chronic administration of these antiepileptic drugs may be associated with significant pharmacokinetic interactions resulting in reducing the plasma concentration of co-administered antidepressants. This predicted interaction was actually confirmed in terms of TCAs and many others–for instance, bupropion, citalopram, escitalopram, paroxetine, reboxetine or venlafaxine (for review, Italiano et al., 2014). For instance, carbamazepine at low daily doses of 200 or 400 mg led to a significant reduction in plasma concentrations of S-citalopram and R-citalopram, by 27 and 31%, respectively (Spina et al., 2016). A comparable effect was observed for paroxetine whose plasma level was decreased by 25% in patients receiving carbamazepine, phenobarbital or phenytoin (Spina et al., 2016). A case-report study (2 patients) even provided evidence that carbamazepine led to a sharp reduction in plasma concentration of sertraline, which was associated with loss of sertraline’s efficacy (Spina et al., 2016). Newer (or second-generation) antiepileptic drugs generally cause less pharmacokinetic interactions with concomitant medications, however, some of them are week enzyme inducers. That is why pharmacokinetic interactions may accompany combinations of clobazam, eslicarbazepine, oxcarbazepine, perampanel, rufinamide, and topiramate combined with antidepressants (Patsalos and Perucca, 2003; Spina et al., 2016). These newer antiepileptics may activate CYP3A4, however, some of them may also behave as weak inhibitors of CYP2C19 (eslicarbazepine, felbamate, oxcarbazepine, topiramate). A possibility of pharmacokinetic interactions with antidepressants is thus much lower than in the case of conventional antiepileptic drugs. The data on this issue are scarce. In 2021, in the single-center, open-label, randomized, 5-period cross-over trial, an interaction between the newer antiepileptic drug, gabapentin (25–300 mg), and trazodone (2.5–10 mg) was evaluated in healthy volunteers following single dose fasted administration. In no case pharmacokinetic interactions were evident in terms of the plasma AUC or C_max_ (Ruggieri et al., 2021).

In contrast to other conventional antiepileptic drugs, valproate is an inhibitor of microsomal liver enzymes, CYP2C9 being distinctly affected. Also, the antiepileptic inhibits UGTs and epoxide hydrolase. It may be thus predicted that valproate can cause increases in concomitant plasma concentrations of antidepressant drugs. This was in fact confirmed in patients taking amitriptyline or venlafaxine. Regarding the latter, the mean, dose-corrected, plasma concentration of its active metabolite, O-desmethylvenlafaxine was elevated by 27% (Italiano et al., 2014; Spina et al., 2016). Among newer antiepileptic drugs, stiripentol inhibits CYP1A2, CYP2C19, and CYP3A4 isoforms (Spina et al., 2016) so potentially, it could affect the pharmacokinetic parameters of antidepressant drugs, however, no data are available on this issue.

## 5 Pharmacokinetic interactions between antidepressant and antiepileptic drugs in clinical conditions

Numerous antidepressants have been documented to inhibit various isoforms of microsomal liver enzymes so these drugs are likely to affect the plasma concentrations of antiepileptics. Theoretically, increases in plasma concentrations of antiepileptic drugs may be expected following concomitant antidepressant drugs inhibiting P450 cytochrome enzymes.

The available evidence points to the fact that this assumption may not necessarily occur. For instance, fluvoxamine (100 mg daily, given for 3 weeks in seven patients), as an inhibitor of CYP1A2 and CYP2C19, did not modify carbamazepine’s (800–1,600 mg daily) plasma concentration. Initial case report studies revealed the existence of a pharmacokinetic interaction between both drugs. Only a case report study is available on the interaction of fluvoxamine with phenytoin, indicating a significant rise in the plasma phenytoin level (Spina et al., 2016). However, fluoxetine which is an inhibitor of CYP2D6 and to a lesser degree inhibiting CYP2C19, caused significant elevations in the plasma levels of a number of antiepileptic drugs—for instance, phenytoin and valproate. In the case of the latter, a probable increase in its toxicity was observed (Monaco and Cicolin, 1999; Spina et al., 2016). There are unequivocal data as regards the influence of fluoxetine on the plasma concentration of carbamazepine. Some case reports point to significant elevations of carbamazepine’s plasma levels but a study conducted on eight patients taking carbamazepine (800–1,600 mg daily revealed no impact of fluoxetine (co-administered for 3 weeks with this AED) on its steady-state concentration (Spina et al., 2016). However, in one case of combined treatment with fluoxetine and carbamazepine, even a Parkinson-like syndrome was reported (Monaco and Cicolin, 1999). Moreover, fluoxetine may elevate the concentration of the carbamazepine’s toxic metabolite, carbamazepine-10,11-epoxide although there are also data reporting no such effect (Monaco and Cicolin, 1999). Equivocal effects were obtained for fluvoxamine and carbamazepine whilst results on fluvoxamine-induced considerable elevation of phenytoin plasma level are only available (Spina et al., 2016; Zaccara and Franco 2022).

Viloxazine co-administered with carbamazepine may lead not only to the rise in the plasma concentration of this AED but also to the increased level of carbamazepine-10,11-epoxide. Whilst the plasma elevation of the AED was in the range of 50% of basal plasma concentration, the metabolite level was increased by 16% (Monaco and Cicolin, 1999). Viloxazine can also affect the metabolism of oxcarbazepine, leading to a 15% rise in the ADE’s metabolite—hydroxycarbazepine and elevate the concentration of phenytoin (Monaco and Cicolin, 1999).

A number of other antidepressant drugs, TCAs (imipramine or nortriptyline) or trazodone may lead to an increase in concentration of phenytoin (Monaco and Cicolin, 1999). Also, trazodone (a CYP3A4 inhibitor) has been shown to precipitate carbamazepine’s neurotoxicity which was evidently associated with the elevated plasma concentration of this AED. Remarkably, the signs of toxicity tended to disappear when the antidepressant drug was withdrawn (Spina et al., 2016).

Interestingly, sertraline (an inhibitor of CYP2D6), although displayed no potential to affect the plasma concentrations of either carbamazepine or phenytoin, it distinctly increases the plasma levels of valproate and lamotrigine, and these combinations were actually associated with toxicity. However, another study did not confirm the interaction of sertraline with lamotrigine (Spina et al., 2016).

Examples of co-administrations of paroxetine and mirtazapine which do not inhibit CYP enzymes involved in the metabolism of AEDs may suggest that the reduced activity of CYP enzymes is generally responsible for the observed interactions between antidepressant drugs and AEDs. Actually, none of these two antidepressants have been reported to significantly affect the plasma levels of AEDs (Monaco and Cicolin, 1999; Spina et al., 2016). Citalopram and escitalopram (as inhibitors of CYP2D6) have been also found not to interact with carbamazepine following chronic administration of both groups of drugs in healthy volunteers (Spina et al., 2016). Pharmacokinetic interactions have been summarized in Tables 3, 4.

**TABLE 3:** Pharmacokinetic interactions between antiepileptic and antidepressant drugs.

Antiepileptic drug	Dose of antiepileptic drugs (mg/kg/day)	Antidepressant; dosage (mg/kg/day)	Plasma concentration of the antidepressant (%)	References
CBZ	200 or 400	Citalopram; 40-60	↓ (21) S-citalopram	Spina et al. (2016)
CBZ	200 or 400	Citalopram; 40-60	↓ (31) R-citalopram	Spina et al. (2016)
CBZ	ND	Paroxetine	↓ (25)	Spina et al. (2016)
CBZ	ND	Sertraline	↓ (ND)	Spina et al. (2016)
GBP	25–300	Trazodone; 2.5-10	0	Ruggieri et al. (2021)
PB	ND	Paroxetine	↓ (25)	Spina et al. (2016)
PHT	ND	Paroxetine	↓ (25)	Spina et al. (2016)
VPA	ND	Amitriptyline	↑ (50-60)	Italiano et al. (2014)
VPA	ND	Venlafaxine	↑ (27)	Spina et al. (2016)

↑- increased; ↓ - decreased; ND, no data; 0–no effect; CBZ, carbamazepine; GBP, gabapentin; PB, phenobarbital; PHT, phenytoin; VPA, valproate.

**TABLE 4:** Pharmacokinetic interactions between antidepressant and antiepileptic drugs.

Antidepressant	Dose of an antidepressant (mg/kg/day)	Duration of treatment with an antidepressant	Antiepileptic drug; dosage (mg/kg/day)	Duration of treatment with an antiepileptic drug	Plasma concentration of antiepileptic drugs (% increase where applicable)	References
Citalopram	40	14 days	CBZ; 400	35 days	0	Spina et al. (2016)
Escitalopram	ND	ND	CBZ; 400	ND	0	Spina et al. (2016)
Fluoxetine	ND	ND	PHT and VPA	ND	↑	Monaco and Cicolin, (1999); Spina et al. (2016)
Fluoxetine	20	3 weeks	CBZ; 800-1,600	3 weeks	0	Spina et al. (2016)
Fluvoxamine	100	3 weeks	CBZ; 800-1,600	3 weeks	0	Spina et al. (2016)
	100	3 weeks	PHT	ND	↑	Spina et al. (2016); Zaccara and Franco (2022)
Imipramine Nortriptyline Trazodone	ND	ND	PHT	ND	↑	Monaco and Cicolin, (1999)
Sertraline	200	17 days	PHT; 300	24 days	0	Spina et al. (2016)
Sertraline	200	17 days	CBZ; 400	32 days	0	Spina et al. (2016)
Sertraline	100	ND	VPA	ND	↑ (3-fold elevation in serum concentration)	Spina et al. (2016)
Sertraline	25	ND	LTG	ND	↑ (2-fold elevation in plasma)	
Viloxazine	ND	ND	CBZ	ND	↑ (up to 50%)	Monaco and Cicolin, (1999)

↑- increased; ↓—decreased; ND, no data; 0–no effect; CBZ, carbamazepine;; LTG, lamotrigine; PHT, phenytoin; VPA, valproate.

## 6 Possibly pharmacodynamic interactions between antiepileptic and antidepressant drugs in clinical conditions

Data on possibly pharmacodynamic interactions between these two groups of drugs are scarce. Kagawa et al. (2014) evaluated the efficacy of lamotrigine in patients with treatment-resistant depressive disorder who were taking 3 psychotropic drugs (antidepressants, mood stabilizers, atypical antipsychotics). Lamotrigine was given at 100 mg daily for 18 patients (without valproate) and 75 mg daily for those taking valproate (*N* = 16). Percentage improvements at week eight was clearly dependent on plasma lamotrigine concentrations of ≥12.7 μmol/L. At this lamotrigine concentration 73% of patients were improved whilst in patients with lower lamotrigine plasma concentrations only 23% benefitted from lamotrigine augmentation. Interestingly, neither serum brain-derived neurotrophic factor nor interleukin-6 were not affected by lamotrigine combined with other antipsychotropic drugs (Kagawa et al., 2017).

Also topiramate and pregabalin were documented to potentiate the antidepressant efficacy of antidepressants (for instance, SSRIs) (Elger et al., 2017).

## 7 Adverse effects associated with co-administration of antiepileptics and antidepressants in patients with epilepsy and depression

One of the adverse effects, in patients taking AEDs and antidepressant drugs, may be associated with body weight gain. Reduced weight gain has been shown to accompany combinations of zonisamide or topiramate with amitryptiline, bupropion, mirtazapine or paroxetine whilst increased weight gain—combinations of carbamazepine, gabapentin, pregabaline or VPA with amitryptiline, mirtazapine or paroxetine (Italiano et al., 2014).

AEDs have been documented to exert a negative impact on bone health and in case, antidepressants possess a similar activity, the combined treatment may eventually lead to additive or even synergistic effects (Miziak et al., 2019). Although, the experimental data do not seem to support such a possibility, the results from two meta-analyses are not that optimistic. Actually, TCAs and SSRIs have been shown to significantly increase the risk fractures (Miziak et al., 2019).

Both some AEDs and antidepressants have been documented to stimulate the activity of CYP enzymes (Italiano et al., 2014). Consequently, it is advised to avoid combined treatments with liver enzyme enhancers among these groups of drugs. This restriction may apply to carbamazepine, felbamate or phenytoin and bupropion, duloxetine, TCAs or trazodone, on the other. These combinations may result in subsequent liver injury (Italiano et al., 2014).

Patients with the presence of long QTc syndrome and family history of sudden death are prone to lethal ventricular arrhythmias (torsade de pointes). Some antidepressants (citalopram, fluoxetine, sertraline) and the AEDs (felbamate and probably carbamazepine, lamotrigine, and phenytoin) (Auerbach et al., 2018) predispose patients to arrhythmias so there combined use is not recommended (Italiano et al., 2014).

Some other adverse effects may accompany combined treatment with AEDs and antidepressants—reduced production of sweat and hypohydrosis, increased risk for hyponatremia (especially when carbamazepine is co-administered with antidepressants) (Banach et al., 2016).

## 7 What is the significance of animal data pointing to the fact that some antidepressants may reduce the anticonvulsant activity of some antiepileptic drugs?

One of reasons for co-administration of AEDs with antidepressants is co-morbid depression in patients with epilepsy although, as already mentioned, it may be frequently underdiagnosed or untreated because of fear of untoward drug interactions (Kanner et al., 2012; Bosak et al., 2015). The existing experimental evidence on pharmacodynamic interactions between AEDs and antidepressants may give possible clues on which drug combinations in depressed patients with epilepsy need to be avoided. The importance of pharmacokinetic interactions between these groups of drugs in experimental animals possess rather no clinical implications due to different metabolic pathways in rodents and humans. However, their estimation in experimental studies could help delineate pure pharmacodynamic or mixed pharmacodynamic/pharmacokinetic interactions. Certainly, pure pharmacodynamic interactions would have more predictive value when transferring the experimental data to clinical conditions.

Theoretically, antidepressant drugs raising the convulsive threshold would seem safe to be combined with AEDs in patients with epilepsy. However, antidepressants start to exert therapeutic effects following chronic administration. That is why they were evaluated in terms of their influence upon the convulsive threshold both after acute and chronic treatment. Mianserin is a good example that acute vs. chronic administration may yield opposite effects—whilst given acutely, it increased the anticonvulsive threshold in mice but its chronic administration resulted in an opposite effect. Comparably, its acute combinations with phenytoin or valproate led to the potentiation of their protective efficacy against maximal electroshock-induced convulsions in mice and chronic ones resulted in significant reductions in their anticonvulsant activities (Borowicz et al., 2007). Chronic trazodone significantly elevated the electroconvulsive threshold in mice and no effect on the threshold was observed after its acute injection (Borowicz et al., 2012a). Interestingly, following its chronic combinations with phenobarbital or carbamazepine, reductions in the anticonvulsant effects of these AEDs against maximal electroshock in mice were evident. Nevertheless these reductions were associated with significant decreases in the brain concentration of these AEDs so, probably, a possibility of pharmacodynamic interactions is minimal. On the other hand, chronic trazodone was neutral upon the protective effect of valproate although its brain concentration was elevated by more than 30%. A possibility arises that the negative impact of trazodone on the anticonvulsant potential of valproate was actually masked by the pharmacokinetic interaction (Borowicz et al. 2012b). Tianeptine (acutely or chronically), although ineffective upon the electroconvulsive threshold, it significantly enhanced the protection offered by carbamazepine and valproate in the chronic protocol, without affecting their brain concentrations (Borowicz et al., 2013). The last example concerns venlafaxine which acutely and chronically raised the electroconvulsive threshold and following its chronic administration, also potentiated the anticonvulsant activity of valproate against maximal electroshock. The protection of other AEDs (carbamazepine, phenobarbital, phenytoin) was not modified (Borowicz et al., 2011).

To the degree, the experimental data may be transferred to clinical conditions, interactions between AEDs and antidepressants resulting in neutral outcomes or in the potentiation of anticonvulsant activity of an AED can be considered safe as regards the seizure control. In contrast, when an antidepressant leads to reduced anticonvulsant effects of AEDs then its clinical use may be not recommended in patients with epilepsy and depression. A neutral effect of an antidepressant masked by a pharmacokinetic interaction, showing a significant rise in the brain concentration of an AED, must be considered negative. When a neutral effect of the combined treatment is associated with a reduced brain concentration of an AED then probably no reduction in seizure control can be expected.

## 8 Conclusion

Mesial temporal lobe epilepsy with hippocampal sclerosis is frequently associated with a prevalence of major depression in the range of up to 25%, which further points to a close pathophysiological relationship between these two disorders (da Nobrega Marinho et al., 2022). The patients with either epilepsy or epilepsy with major depression exhibited a reduced expression of 5-HT_1A_ receptors in comparison with the control patients (da Nobrega Marinho et al., 2022). Possibly, SSRIs would be a good choice to treat major depression in these patients considering that these drugs did not negatively modify the anticonvulsant activity of a number of AEDs in experimental conditions.

Chronic venlafaxine, both elevated the threshold and enhanced the anticonvulsant activity of valproate although other conventional AEDs were not affected. Mianserin reduced the threshold and the protective activity of valproate and phenytoin. Anyway, on the basis of the effects of antidepressant drugs upon the convulsive threshold, it is not possible to predict their interactions with AEDs. Actually, although chronic trazodone increased the convulsive threshold, its interactions with AEDs were assumed negative. Another example also shows that a chronic antidepressant without effect on the threshold, can effectively potentiate the anticonvulsant activity of AEDs (tianeptine and carbamazepine or valproate). Even though, experimental data may yield significant clues to the clinicians on the pharmacodynamic interactions between AEDs and antidepressants, patients with epilepsy and depression require careful monitoring for seizure frequency and adverse effects. Preclinical findings (mainly from studies with maximal electroshock-induced convulsions in mice) indicate that safe antidepressants for patients with epilepsy might be bupropion, fluoxetine, milnacipran, moclobemide, paroxetine, reboxetine, tianeptine, and venlafaxine. On the other hand, results of experimental studies may discourage using mianserin (especially in patients with epilepsy on phenytoin or valproate) and trazodone (in patients receiving valproate). The clinical evidence is generally in accordance with experimental data on safe antidepressants in patients with epilepsy (Harden and Goldstein, 2002), the exception being bupropion reported as a drug with a greater risk of seizures (Harden and Goldstein, 2002; Pisani et al., 2022). Other antidepressants to be used with caution include clomipramine and maprotiline (Harden and Goldstein, 2002; Pisani et al., 2022). No clinical support exists for mianserin and trazodone as antidepressants not recommended in patients with epilepsy.

In an elegant review on many experimental aspects of the interactions between AEDs and antidepressant drugs, Borowicz-Reutt (2021) has analyzed probable mechanisms involved in the potentiation of the anticonvulsant activity of some AEDs by antidepressant co-treatment. One of such mechanisms might be reduced synaptic glutamate release (fluoxetine, reboxetine, venlafaxine). Probably, the brain-derived neurotrophic factor (BDNF) could be involved, considering that some antidepressants were documented to increase its brain expression (for example, moclobemide or fluoxetine) and BDNF itself exhibited its own anticonvulsant activity (Borowicz-Reutt, 2021). Also, enhanced serotonergic, noradrenergic or dopaminergic neurotransmission by a number of antidepressants could be involved in the positive interactions between these groups of drugs as generally, stimulation of at least some serotonergic, noradrenergic or D-2 dopaminergic receptors was found anticonvulsant in some experimental models of seizures (Löscher and Czuczwar, 1985; Löscher and Czuczwar, 1986; Löscher and Czuczwar, 1987).

Mianserin, as a drug increasing the noradrenergic action (Dell’Osso et al., 2011), was found to reduce the anticonvulsant activity of phenytoin and valproate which is hard to explain at the moment. Probably, chronic administration of this antidepressant resulted in a number of events leading eventually to negative pharmacodynamic interactions with these AEDs. A similar situation was encountered when analyzing the effect of calcium channel inhibitors upon the anticonvulsant activity of AEDs against maximal electroshock-induced seizures in mice. Although calcium channel inhibitors generally enhanced the anticonvulsant activity of AEDs (Kułak et al., 2004), niguldipine was an exception to the rule. Whilst it elevated the electroconvulsive threshold, but when combined with AEDs, it led to the reduction of the anticonvulsant action of carbamazepine and phenobarbital (Borowicz et al., 1997).

As already mentioned, AEDs and antidepressants may interact via pharmacokinetic or pharmacodynamic mechanisms. The available data on the efficacy of antidepressants in alleviating depressive symptoms associated with epilepsy, however, seems limited. According to Maguire et al. (2021), who have reviewed randomized controlled trials and non-randomized studies of interventions, responses to antidepressants in patients with epilepsy were highly variable. Possibly, better results may be achieved when a patient is prescribed carbamazepine, gabapentin, lamotrigine, topiramate or valproate. These antiepileptics have been documented to improve mood (Alhashimi et al., 2022; Shamabadi, 2022).

Finally, as already mentioned, polytherapy in epilepsy is a recognized risk factor for the development of depression. The existing experimental data on interactions between AEDs and antidepressants refer to one drug from each group so no experimental clues are available on this issue. Therefore, management of depression in patients with epilepsy on polytherapy seems more challenging and may result in more negative drug interactions.
